# Whole exome sequencing in ADHD trios from single and multi-incident families implicates new candidate genes and highlights polygenic transmission

**DOI:** 10.1038/s41431-020-0619-7

**Published:** 2020-04-01

**Authors:** Bashayer R. Al-Mubarak, Aisha Omar, Batoul Baz, Basma Al-Abdulaziz, Amna I. Magrashi, Eman Al-Yemni, Amjad Jabaan, Dorota Monies, Mohamed Abouelhoda, Dejene Abebe, Mohammad Ghaziuddin, Nada A. Al-Tassan

**Affiliations:** 1 Behavioral Genetics unit, Department of Genetics, King Faisal Specialist Hospital and Research Center, P.O Box 3354, Riyadh, 11211 Saudi Arabia; 2 Saudi Human Genome Program, King Abdulaziz City for Science and Technology, Riyadh, Saudi Arabia; 3 National center for genomics technology, King Abdulaziz City for Science and Technology, Riyadh, Saudi Arabia; 4 Department of Genetics, King Faisal Specialist Hospital and Research Center, P.O Box 3354, Riyadh, 11211 Saudi Arabia; 5 Systems and Biomedical Engineering Department, Faculty of Engineering, Cairo University, Giza, Egypt; 6 Psychiatry Department, King Faisal Specialist Hospital and Research Center, P.O Box 3354, Riyadh, 11211 Saudi Arabia; 7 University of Michigan, Ann Arbor, MI USA

**Keywords:** ADHD, Next-generation sequencing

## Abstract

Several types of genetic alterations occurring at numerous loci have been described in attention deficit hyperactivity disorder (ADHD). However, the role of rare single nucleotide variants (SNVs) remains under investigated. Here, we sought to identify rare SNVs with predicted deleterious effect that may contribute to ADHD risk. We chose to study ADHD families (including multi-incident) from a population with a high rate of consanguinity in which genetic risk factors tend to accumulate and therefore increasing the chance of detecting risk alleles. We employed whole exome sequencing (WES) to interrogate the entire coding region of 16 trios with ADHD. We also performed enrichment analysis on our final list of genes to identify the overrepresented biological processes. A total of 32 rare variants with predicted damaging effect were identified in 31 genes. At least two variants were detected per proband, most of which were not exclusive to the affected individuals. In addition, the majority of our candidate genes have not been previously described in ADHD including five genes (*NEK4*, *NLE1*, *PSRC1*, *PTP4A3,* and *TMEM183A*) that were not previously described in any human condition. Moreover, enrichment analysis highlighted brain-relevant biological themes such as “Glutamatergic synapse”, “Cytoskeleton organization”, and “Ca^2+^ pathway”. In conclusion, our findings are in keeping with prior studies demonstrating the highly challenging genetic architecture of ADHD involving low penetrance, variable expressivity and locus heterogeneity.

## Introduction

The World health organization (WHO) estimates (10–20%) of children worldwide to be affected with some form of childhood-onset neuropsychiatric disorder [https://www.who.int/mental_health/maternal-child/child_adolescent/en/]. These disorders, if left unmanaged, can impair normal functioning and development. Perhaps the most common example of such conditions is attention deficit hyperactivity disorder (ADHD), characterized by a persistent pattern of inattention and/or hyperactivity–impulsivity. In some cases, these symptoms continue into adulthood [1].

Like other neuropsychiatric disorders (e.g., autism, schizophrenia, and depression), the exact causes of ADHD are not yet clearly understood. The earliest evidence for genetic contribution to ADHD came from family, twin and adoption studies consistently demonstrating high heritability (up to 88%) [2]. While this disorder may aggregate in families, it tends not to segregate in a typical Mendelian manner. This is true not only for ADHD, but also for most of complex disorders, whereby risk to the disorder is several folds higher in the relatives of the proband compared with the general population. Also, disease risk was found to be proportional with the degree of relatedness [3, 4]. The current consensus is that ADHD is a complex condition influenced by multiple genetic, social, and environmental factors [5].

Various genome-wide approaches have been employed to investigate the genetic basis of ADHD. For a comprehensive review see [2, 6]. The first wave of studies applied genetic linkage methods on sibling pairs, small families, or multigenerational families in search for genetic variants with large effect. Because of the high prevalence of ADHD (possibly due to increased recognition of the disorder), later studies focused on finding common single nucleotide polymorphisms (SNPs) with modest effects that are significantly associated with the disorder in case-control samples. In these studies one of two main strategies were adopted: candidate gene association or genome-wide association. The former strategy is hypothesis-driven, whereby the selection of candidate genes is based on existing knowledge about; (1) their relevant biological roles, (2) their participation to a recognized ADHD drug target pathway, and (3) their previous association with ADHD. Results from this type of studies were often inconsistent for the same locus. However, only through genome-wide association studies (GWAS) meta-analysis that researchers were able to identify significant associations across studies [7].

Unlike candidate gene association studies, GWAS are hypothesis-free interrogation of the entire genome for significant risk loci (SNPs). Previous GWAS of ADHD failed to find variants with genome-wide significance even when they were combined in a meta-analysis. This is expected given the highly stringent *P* value threshold (5 × 10^−8^) required in this approach, which can only be achieved using a very large sample size [8]. Thus far, only a single study was able to detect association signal with global significance [7]. In this well-powered study (20,183 cases and 35,191 controls), Demontis et al. identified 12 independent risk loci that were also found by another group to be a robust set from which polygenic risk scores could be derived for reliable prediction of some of ADHD concurrent traits [9].

Although up to one-third (estimates ranging from 0.1 to 0.28) of total ADHD heritability is thought to be based on inherited common SNPs, a large portion of the heritable risk remains unaccounted for [4, 10]. This so-called “missing heritability” is likely to be, at least partially, explained by rare genetic variants. The first attempts to address the role of rare variants in ADHD susceptibility were focused on studying copy number variants (CNVs). While enrichment for large CNVs (≥100 kbp) has been observed in a couple of studies [11, 12], the majority of the reported CNVs had reduced penetrance and limited replication across studies [2, 13]. Only recently, have next generation sequencing (NGS) research begun to explore the role of another class of rare variants (single nucleotide variants (SNVs)) in ADHD. However, a very limited number of studies have been published so far [14–19]. In all of these studies exome sequencing was applied for identification of rare functional SNVs either in preselected set of genes [14–16] or across the genome in a small sample size [16–19].

Collectively around 359 genes (counts from ADHDgene database, http://adhd.psych.ac.cn/index.do [accessed on 17.12.19] [20]) have been published in ADHD genetic studies. Most of these studies were conducted in Asian or European populations. The increasing realization of the disadvantages of lack of diversity calls for new diversity-increasing strategies to minimize bias and ensure worldwide applicability of the genetic findings. Such strategies are the path for realizing the promise of personalized medicine for all. The present study contributes to the growing efforts to enrich diversity by including an underrepresented non-European population. We report here the first attempt to explore the role of inherited and de novo variants (SNVs and CNVs) in ADHD (parents–child) trios of Arab descent. Using whole exome sequencing (WES), we applied our in-house analysis pipeline to identify potential ADHD risk variants.

## Subjects and methods

### Participants

A total of 16 Saudi families with at least one child affected with ADHD were recruited in this study after obtaining signed informed consent in accordance with the Declaration of Helsinki and the institution’s relevant committees; Institutional Review Board (IRB), Research Ethics committee and Basic Research Committee. All experimental protocols used in this study were authorized under an IRB-approved project (RAC#2120001). Recruitment was done through the psychiatry department at King Faisal Specialist Hospital and Research Centre (KFSHRC). All of the approached families agreed to participate (no withdrawals). Male to female ratio of the enrolled cases was 3:1 and the mean age was 12 years (Table 1). Probands were assessed by a trained medical team (child psychiatrist and neurologist) and diagnosed according to the diagnostic and statistical manual of mental disorders (DSM-IV) criteria. Our exclusion criteria included ADHD secondary to syndromes with known genetic causes such as Fragile X syndrome, Tuberous Sclerosis, Rett syndrome, Angelman, Prader-Willi syndrome, or Phenylketonuria. Therefore, only cases with non-syndromic ADHD were selected. Blood samples for DNA extraction, were collected from all available consenting family members (parents as well as affected and unaffected siblings).

**Table 1:** Age and gender distribution of enrolled ADHD cases.

Family-ID	Affected individuals	Gender	Age at recruitment (years)
F1	P	M	17
	AS	M	19
	AS	M	13
	AS	M	7
F2	P	M	8
F5	P	M	11
F7	P	M	16
	AS	M	17
F9	P	M	13
	AS	F	17
F10	P	F	16
	AS	M	21
F12	P	F	11
F14	P	M	9
F15	P	F	8
F16	P	M	6
F17	P	F	12
	AS	M	8
F19	P	M	10
	AF	M	NA
F21	P	M	10
	AS no DNA	F	NA
F22	P	M	15
	AS	M	14
	AS	M	10
	AS	M	5
F24	P	M	9
F25	P	M	16
	AS	M	14
	AS	M	13
	AS^a^	F	4

*P* proband, *AF* affected father, *AS* affected sibling, *M* male, *F* female, *NA* not available or not applicable.

^a^Suspected ADHD case.

### Copy number variation analysis

All recruited samples were surveyed for CNVs in genes listed in (Table S1). The list contains genes from AutismKB core dataset (with a total score ≥20), International Multisite ADHD Genetics (IMAGE) candidate risk genes [21] and genes participating in neurotransmitter systems that were not included in the aforementioned sets. CytoScan^TM^ HD Suite (Affymetrix, Santa Clara, CA, USA) was used for genome-wide detection of CNVs. Targeted analysis of CNVs in the selected genes was performed using Chromosome Analysis Suite 3.0 (ChAS 3.0). All genomic locations were based on GRCh37/hg19 human genome assembly. Gains were defined as (log2 ratio of copy) values greater than 0.58 and loss as values less than −1.

### Whole exome sequencing and data analysis

**Fig. 1:**
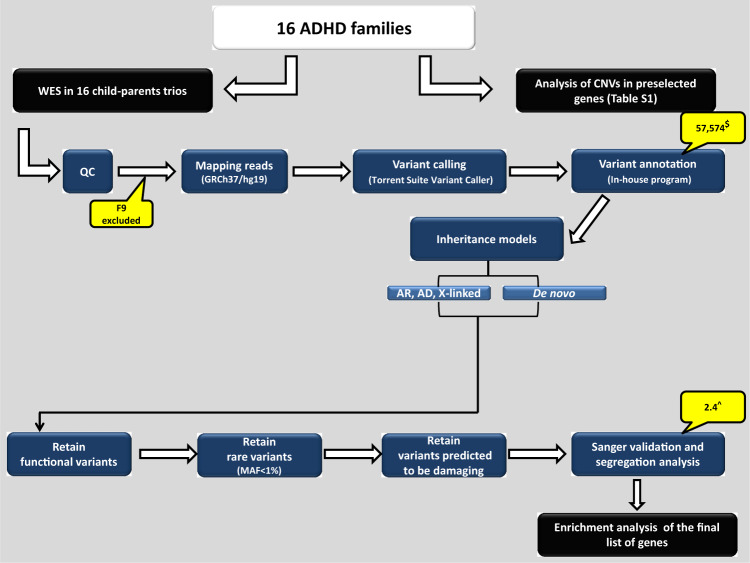
Schematic illustration of the study design and analysis workflow. F9 trio did not pass QC check therefore; it was excluded from further analysis. Dollar symbol represents the average variant count per sample. Hat symbol represents the average number of validated prioritized variants per proband.

The exomes of 16 (parents–child) trios were captured using whole exome AmpliSeq kit and sequenced on Ion Proton^TM^ System platforms (up to 200 bp, single reads). NGS raw data are deposited at the Saudi Human Genome Program (SHGP) repository [https://genomics.saudigenomeprogram.org/en/]. All variants described in this study have been submitted to LOVD [https://databases.lovd.nl/shared/genes] public repository. Sample processing and all three stages of data analysis (primary, secondary, and tertiary) were performed as previously described [22]. The WES workflow applied in this study is summarized in Fig. 1.

#### Variants filtering, validation, and prioritization

Variants obtained by applying the four possible modes of inheritance (autosomal recessive, autosomal dominant, X-linked, and de novo) were retained if present in genes with positive brain expression in three out of four databases; GTEx [23], BioGPS [24], CGAP [25], and human brain transcriptome (HBT) [26]. Next, only functional variants (missense, small insertions/deletions (indels) and loss of function (LoF)) with minor allele frequency (MAF) of <1%) in population databases (1000 Genomes project, ExAC and Kaviar) and in the local ethnically matching Saudi Human Genome Program normal controls database (SHGPdb >2379 exomes) were selected for Sanger validation. The resulting variants were prioritized based on their functional effect prediction whereby only those predicted to be “deleterious” by at least two out of three bioinformatics tools (CADD, PredictSNP2 and FATHMM-MKL) [27–29] were considered. At the gene level, intolerance to variation (missense and LoF) metrics (pLI and Z score) were extracted for each identified gene with validated variant(s). However, although these metrics can be helpful when combined with other prediction tools, they are inadequate to infer or exclude pathogenicity when used solely [22]. In addition, publicly available variants or gene databases such as dbSNP, SFARI Gene [https://gene.sfari.org/autdb/Welcome.do], AutismKB [http://autismkb.cbi.pku.edu.cn/], and ADHDgene [http://adhd.psych.ac.cn/] were checked for previous reports of the variants/genes identified herein. When possible, segregation analysis was performed on affected as well as unaffected siblings.

#### Relatedness assessment

Relationships between samples were computed using two algorithms. First we utilized VCFtools program package (with option-relatedness) to calculate the “unadjusted” A_jk_ relatedness statistic based on Yang et al. method. In the second method, shared homozygosity was calculated by comparing the homozygous variants (MAF > 1%) between each pair of individuals and counting the number of overlapping variants after normalization. For detailed description refer to the supplementary material.

#### Functional enrichment analysis

Two enrichment analysis tools were employed to discover biological processes overrepresented in our gene list. The first was WEB-based Gene Set Analysis Toolkit (WebGestalt) [http://www.webgestalt.org] which serves as an integrated data mining system for functional enrichment analysis of large sets of genes. For detailed description refer to the supplementary material. In addition to WebGestalt, we utilized the Reactome Knowledgebase [www.reactome.org] which functions as an archive for biomolecular pathways besides offering overrepresentation data analysis.

## Results

### Analysis of CNVs

None of the genes included in our list (Table S1) were harbored in the regions with copy number alterations except for a heterozygous copy loss in *SHANK3* that was identified in probands from F5 and F15. The deleted region spans the entire gene in F5, whereas in F15 it was found to overlap with parts of *SHANK3*. Of note, our analysis revealed CNVs in two genes that were not included in our gene list but are worth reporting here given their reported link to autism spectrum disorder (ASD) [30, 31]. The first was *NOTCH1*, in which copy gain was detected in probands of F10 and F17. The second was *NRXN3* that was found to harbor heterozygous copy loss in F19. Locations and details of the detected CNVs are listed in Table 2

**Table 2:** CNVs detected in this study.

Family-ID	Family members	Affected region								Ensembl genome browser						
		Location (hg19) CytoscanHD nomenclature	Location (hg19) HGVS nomenclature^a^	Start	End	Size (Kbp)	CN status	Gene count	Within known CNV^b^	Gene of interest	Physical location^c^	Physical location^d^ (sequence direction)	Transcript ID	Exons order (numbering)	Exons harboring the CNV start-end (physical location)	Introns harboring the CNV start-end (physical location)
F5	P (M)	arr[hg19] 22q13.33(51,073,379–51,197,838)×1	NC_000022.10:g.(?_51073379)_(51197838_?)del	51,073,379	51,197,838	124.459	Loss (het)	3	Y	*SHANK3*	chr22:51,113,070–51,171,640	chr22: 51,113,070–51,171,641 forward strand	NM_033517/ENST00000262795.3	1–23	Entire gene	Entire gene
	US (F)	arr[hg19] 22q13.33(51,073,379–51,197,725)×1	NC_000022.10:g.(?_51073379)_(51197725_?)del	51,073,379	51,197,725	124.346	Loss (het)	3	Y							
	US (M)	arr[hg19] 22q13.33(51,130,158–51,183,869)×1	NC_000022.10:g.(?_51130158)_(51183869_?)del	51,130,158	51,183,869	53.711	Loss (het)	3	Y							
	UM	arr[hg19] 22q13.33(51,073,379–51,197,838)×1	NC_000022.10:g.(?_51073379)_(51197838_?)del	51,073,379	51,197,838	124.459	Loss (het)	3	Y							
	UF	arr[hg19] 22q13.33(51,073,265–51,197,838)×1	NC_000022.10:g.(?_51073265)_(51197838_?)del	51,073,265	51,197,838	124.573	Loss (het)	3	Y							
F10	P (F)	arr[hg19] 9q34.3(139,252,010–139,435,356)×3	NC_000009.11:g.(?_139252010)_(139435356_?)dup	139,252,010	139,435,356	183.346	Gain	11	Y	*NOTCH1*	chr9:139,388,896–139,440,238	chr9: 139,388,896–139,440,314 reverse strand	NM_017617/ENST00000277541.6	1–34	From exon 3 to exon 34 139,418,431–139,388,896	From intron (2–3) to (33–34) 139,438,475–139,392,011
	AS (M)	No change	–	–	–	–	–	–	–							
	UF	No change	–	–	–	–	–	–	–							
	UM	arr[hg19] 9q34.3(139,114,307–139,435,356)×3	NC_000009.11:g.(?_139114307)_(139435356_?)dup	139,114,307	139,435,356	321.049	Gain	13	Y							
F15	P (F)	arr[hg19] 22q13.33(51,127,896–51,197,725)×1	NC_000022.10:g.(?_51127896)_(51197725_?)del	51,127,896	51,197,725	69.829	Loss (het)	3	Y	*SHANK3*	chr22:51,113,070–51,171,640	chr22: 51,113,070–51,171,641 forward strand	NM_033517/ENST00000262795.3	1–23	From exon 10 to exon 23 51,133,203–51,160,865	Parts of intron (9–10) to intron (22–23) 51,123,080–51,160,865
	US (M)	No change	–	–	–	–	–	–	–							
	US (F)	No change	–	–	–	–	–	–	–							
	US (F)	No change	–	–	–	–	–	–	–							
	UM	No change	–	–	–	–	–	–	–							
	UF	No change	–	–	–	–	–	–	–							
F17	P (F)	arr[hg19] 9q34.3(139,375,930–139,435,356)×3	NC_000009.11:g.(?_139375930)_(139435356_?)dup	139,375,930	139,435,356	59.426	Gain	4	Y	*NOTCH1*	chr9:139,388,896–139,440,238	chr9: 139,388,896–139,440,314 reverse strand	NM_017617/ENST00000277541.6	1–34	From exon 3 to exon 34 139,418,431–139,388,896	From intron (2–3) to (33–34) 139,438,475–139,392,011
	AS (M)	No change	–	–	–	–	–	–	–							
	US (M)	No change	–	–	–	–	–	–	–							
	US (M)	arr[hg19] 9q34.3(139,053,501–139,654,647)×3	NC_000009.11:g.(?_139053501)_(139654647_?)dup	139,053,501	139,654,647	601.146	Gain	27	Y							
	UM	No change	–	–	–	–	–	–	–							
	UF	arr[hg19] 9q34.3(139,056,879–139,431,947)×3	NC_000009.11:g.(?_139056879)_(139431947_?)dup	139,056,879	139,431,947	375.068	Gain	14	Y							
F19	P (M)	arr[hg19] 14q31.1(79,342,954–79,397,500)×1	NC_000014.8:g.(?_79342954)_(79397500_?)del	79,342,954	79,397,500	54.546	Loss (het)	1	Y	*NRXN3*	chr14:78,870,074–80,334,633	chr14: 78,870,093–80,328,786 forward strand	NM_004796/ENST00000554719.1	1–17	NA	Parts of intron (7–8) 79,276,688–79,423,584
	US (F)	arr[hg19] 14q31.1(79,342,954–79,401,388)×1	NC_000014.8:g.(?_79342954)_(79401388_?)del	79,342,954	79,401,388	58.434	Loss (het)	1	Y							
	US (F)	arr[hg19] 14q31.1(79,342,954–79,397,500)×1	NC_000014.8:g.(?_79342954)_(79397500_?)del	79,342,954	79,397,500	54.546	Loss (het)	1	Y							
	AF	No change	–	–	–	–	–	–	–							
	UM	arr[hg19] 14q31.1(79,342,954–79,401,337)×1	NC_000014.8:g.(?_79342954)_(79401337_?)del	79,342,954	79,401,337	58.383	Loss (het)	1	Y							

*P* proband, *UM* unaffected mother, *AF* affected father, *AS* affected sibling, *UF* unaffected father, *M* male, *F* female.

^a^Information about deletion/duplication breakpoints were not available nor the position of the first or last normal nucleotides. Therefore CNV description was based on HGVS recommendations for describing variants for which not all details are available.

^b^CNVs identified here that are wholly or partially contained within regions reported in the Database of Genomic Variants (DGV) [http://dgv.tcag.ca/dgv/app/home].

^c^Genes positions are based on UCSC Genome Browser on Human Feb. 2009 (GRCh37/hg19) Assembly.

^d^Physical location based on Ensembl Genome browser.

### WES data, performance, and statistics

Results of relatedness assessment and demographic information are summarized in Table S2. Performance metrics of the WES runs including, number of reads per base that map to the reference genome, the percentage of target region coverage and sequencing depth are summarized in Table S3. High performance metrics were achieved indicating high quality mapping and variant calling. Type and number of the detected variants before applying the filtering pipeline are listed in Table S4. All trios passed quality control checks except for F9 which was excluded from further analysis.

### Rare SNVs detected by WES

By interrogating all protein coding regions covered by the employed kit, we have identified and validated a total of 32 unique rare variants in 31 different genes. Of which, 2 were de novo and 30 were inherited mainly being missense changes. Interestingly, 5 of the identified genes (*NEK4*, *NLE1*, *PSRC1*, *PTP4A3*, and *TMEM183A*) have not been previously described in any human disorder. The biological function of 10/31 genes is yet to be determined, while others participate in similar biological themes. For instance, signal transduction (*THBS4*, *PLCB2*, *GNB4*, *PTCH1*), chromatin modeling or DNA damage response (*NEK4*, *ACIN1*, *PSRC1*, *TLK2*, *SPAG5*), gene transcription regulation (*KMT2A* and *ZFHX3*), and vesicle transport (*NBAS*, *KIF21A*, *PCLO*) (Table S5).

Positive brain expression was reported for all the identified genes, also information on the developmental and regional differential expression was obtained from HBT (Table S6). Information was available for 26/32 identified genes, 12 of which were expressed throughout the entire life span, 13 were under temporal and/or spatial control, and 1 gene (*ACCS*) had a negative expression in the brain. *NLE1* is the only gene that appears to be developmentally regulated across all included brain regions. Based on the HBT database, its expression is restricted to a limited developmental time frame (embryonic—late mid fetal development) [26].

The absence of a universal candidate gene/variant for neurodevelopmental disorders like ASD and ADHD is consistent with the increasingly accepted view that heterogeneous set of genetic variants, displaying incomplete penetrance, can shape risk to the disorder [32, 33]. In light of this, we considered a model in which a combination of genetic variants forming a “constellation” may influence risk. Therefore, we have reported all SNVs that have survived our filtering criteria regardless of their co-segregation profile (Table 3). As expected, most of the validated variants exhibited reduced penetrance being either present in normally developing siblings or absent in the affected ones. One exception was *LRRK1* LoF variant (c.2687-2A>G) that was detected in a homozygous state in three out the four affected individuals in F1, while the remaining (screened) family members including the 4th affected sibling were all heterozygous carriers for this change. These variants are an example of variation in penetrance and phenotypic expressivity, a phenomenon that is not uncommon in neurodevelopmental disorders.

**Table 3:** Rare variants detected and validated in this study.

Family-ID	Gene	c.DNA	Protein	SNPdb	Frequency				Prediction tools				Screened individuals-genotypes							
					SHGPdb	1000G	ExAC	Kaviar	CADD(phred)	PredictSNP2 (%)	FATHMM-MKL (score)	ExAC (pLI/Z score)								
**F1**				–									**UM**	**UF**	**P (M)**	AS (M)	AS (M)	AS (M)	US (F)	US (M)
	*ACIN1*	NM_001164816.2:c.214G>T	NP_001158288.1:p.(Asp72Tyr)	–	0.000420345	0	–	–	23.5	Neutral (63)	0.85074	1.00/2.98	HET	HOM	HET	HOM	HOM	HOM	HOM	HOM
	*LRRK1*^a^	NC_000015.9 (NM_024652.6):c.2687-2A>G	–	–	0	0	–	–	33	Deleterious (63)	0.98391	0.0/3.12	HET	HET	HOM	HOM	HET	HOM	HET	HET
**F2**	No candidate variants found																			
**F5**													**UM**	**UF**	**P (M)**	US (F)	US (M)			
	*DIAPH3*^a^	NM_001258366.2:c.2881G>A	NP_001245295.1:p.(Glu961Lys)	–	0.004203447	0	3.39E−05	2.59E−05	21.3	Neutral (63)	0.90278	0.0/−0.42	WT	HET	HET	HET	HET			
	*LIMCH1*	NM_001112720.3:c.1174G>A	NP_001106191.1:p.(Gly392Arg)	–	0.005884826	0	0.0001	7.76E−05	25.3	Deleterious (87)	0.99105	1.00/−0.11	HET	HET	HET	HET	HET			
	*LIMCH1*	NM_001112720.3:c.1942C>T	NP_001106191.1:p.(Arg648Cys)	–	0.001261034	0	1.30E−05	6.50E−06	34	Deleterious (82)	0.85122	1.00/−0.11	HET	WT	HET	HET	WT			
	*NEK4*	NM_001193533.2:c.671C>T	NP_001180462.1:p.(Ser224Phe)	–	0.002101723	0	–	–	21.1	Deleterious (87)	0.93070	0.0/−0.19	HET	WT	HET	WT	WT			
**F7**													**UM**	**UF**	**P (M)**	AS (M)				
	*PCLO*^a^	NM_014510.3:c.10918T>C	NP_055325.2:p.(Phe3640Leu)	–	0.000420345	0	–	–	28.2	Deleterious (82)	0.98562	1.00/−4.32	HET	HET	HOM	WT				
	*ACCS*	NM_001127219.2:c.1370G>A	NP_001120691.1:p.(Arg457His)	–	0.002101723	0	1.65E−05	1.29E−05	26.8	Deleterious (87)	0.97866	0.0/−0.64	WT	HET	HET	WT				
**F10**													**UM**	**UF**	**P (F)**	AS (M)				
	*CRELD2*^a^	NM_001135101.3:c.295G>A	NP_001128573.1:p.(Glu99Lys)	–	0.000420345	0	2.01E−05	1.29E−05	27.5	Deleterious (87)	0.97981	0.0/−0.01	WT	HET	HET	WT				
**F12**													**UM**	**UF**	**P (F)**					
	*THBS4*	NM_003248.6:c.2738A>G	NP_003239.2:p.(Asp913Gly)	–	0.000420345	0	–	–	32	Deleterious (87)	0.95833	0.0/0.85	HET	WT	HET					
	*PDHA1*	NM_001173456.1:c.812G>A	NP_001166927.1:p.(Arg271His)	–	0	0	–	–	33	Deleterious (87)	0.97536	0.99/2.78	WT	WT	HET					
**F14**													**UM**	**UF**	**P (M)**	US (F)	US (M)			
	*PTP4A3*^a^	NM_032611.3:c.241G>A	NP_116000.1:p.(Val81Met)	–	0	0	–	–	25.6	Deleterious (87)	0.96047	0.13/1.62	HET	HET	HOM	HET	HOM			
	*TMEM183A*	NM_138391.6:c.231G>C	NP_612400.3:p.(Gln77His)	–	0.007566204	0	–	–	21.6	Deleterious (87)	0.93121	0.95/2.54	HET	HET	HOM	WT	HOM			
**F15**													**UM**	**UF**	**P (F)**	US (M)	US (F)	US (F)		
	*PPFIBP1*	NC_000012.11(NM_177444.3):c.1633 + 5G>A	–	–	0.001261034	0	0	0	14.39	Deleterious (91)	0.90405	0.0/−0.17	WT	HET	HET	HET	WT	WT		
	*PTCH1*^a^	NM_000264.5:c.3940C>T	NP_000255.2:p.(Pro1314Ser)	–	0.000186116	0.000798722	0.0005	0.0003946	22.6	Deleterious (87)	0.99389	1.00/2.86	HET	WT	HET	HET	HET	HET		
	*ZFHX3*^a,b^	NM_006885.4:c.6685C>G	NP_008816.3:p.(Pro2229Ala)	–	0.009667928	0	3.30E−05	2.59E−05	22.9	Neutral (65)	0.99653	1.00/−1.68	WT	HET	HET	HET	HET	HET		
**F16**													**UM**	**UF**	**P (M)**	US (M)				
	*KMT2A*^a^	NM_001197104.2:c.9962C>T	NP_001184033.1:p.(Thr3321Ile)	–	0.006073598	0.000199681	0.0002	0.0001876	21.9	Neutral (89)	0.85724	1.00/6.64	WT	HET	HET	WT				
	*NLE1*	NM_018096.5:c.313C>T	NP_060566.2:p.(Arg105Cys)	–	0	0	2.47E−05	6.50E−06	28.2	Deleterious (87)	0.95952	0.05/1.07	WT	HET	HET	WT				
**F17**													**UM**	**UF**	**P (F)**	AS (M)	US (M)	US (M)		
	*ACSS3*	NM_024560.4:c.802G>A	NP_078836.1:p.(Gly268Ser)	–	0.000420345	0	–	–	22.2	Deleterious (87)	0.96726	0.0/0.30	HET	WT	HET	WT	HET	HET		
	*SYNE1*^a^	NM_182961.4:c.5843G>T	NP_892006.3:p.(Cys1948Phe)	–	0	0	5.77E−05	5.17E−05	25.6	Deleterious (82)	0.99237	0.0/−0.95	WT	HET	HET	WT	HET	HET		
	*NBAS*^a^	NM_015909.4:c.4692G>T	NP_056993.2:p.(Gln1564His)	–	0	0	2.49E−05	6.50E−06	23.1	Neutral (63)	0.83271	0.0/−3.06	WT	HET	HET	WT	HET	HET		
**F19**													**UM**	**AF**	**P (M)**	US (F)	US (F)			
	*DNAH1*	NM_015512.5:c.8854A>C	NP_056327.4:p.(Ile2952Leu)	–	0	0	–	–	22.1	Neutral (63)	0.93847	0.0/−0.89	WT	HET	HET	HET	WT			
	*PLCB2*^a^	NM_001284297.2:c.1273C>T	NP_001271226.1:p.(Arg425Trp)	rs369261333	0	0	3.32E−05	3.23E−05	17.48	Deleterious (82)	0.88593	0.0/1.53	HET	WT	HET	WT	WT			
	*SRRM2*	NM_016333.4:c.6970C>G	NP_057417.3:p.(Pro2324Ala)	–	0	0	0.0001	0.0001035	22.7	Neutral (89)	0.89484	NA	HET	HET	HET	HET	HET			
	*TLK2*	NM_006852.5:c.2089C>T	NP_006843.2:p.(Arg697Ter)	–	0	0	–	–	39	Deleterious (77)	0.92219	1.00/5.67	WT	WT	HET	WT	WT			
	*SLC9A9*^a^	NM_173653.4:c.1486G>A	NP_775924.1:(p.Asp496Asn)	rs111291437	0.00035727	0.000599042	0.001	0.0009185	23.7	Deleterious (82)	0.87253	0.0/−0.25	HET	WT	HET	HET	HET			
**F21**													**UM**	**UF**	**P (M)**					
	*GNB4*	NM_021629.4:c.668C>T	NP_067642.1:p.(Tyr223Met)	rs144385061	0	0.000399361	0.0002	0.0001423	24.5	Deleterious (87)	0.96820	0.63/1.90	HET	HET	HOM					
	*SPAG5*^a^	NM_006461.4:c.3049C>T	NP_006452.3:p.(Gln1017Ter)	–	0	0	–	–	36	Deleterious (57)	0.81069	0.0/0.08	WT	HET	HET					
	*DOPEY2*/*DOP1B*^a^	NC_000021.8(NM_005128.3):c.2775 + 5G>C	–	–	0.002522068	0	–	–	15.00	Deleterious (97)	0.99276	0.0/0.22	HET	HET	HOM					
**F22**													**UM**	**UF**	**P (M)**	AS (M)	AS (M)	AS (M)		
	*HEATR1*	NM_018072.6:c.3803C>T	NP_060542.4:p.(Pro1268Leu)	–	0.000840689	0	–	–	24.0	Deleterious (87)	0.94516	1.00/−1.13	WT	HET	HET	WT	WT	HET		
**F24**	No candidate variants found																			
**F25**													**UM**	**UF**	**P (M)**	AS (M)	AS (M)	AS (F)^c^	US (M)	US (M)
	*PSRC1*	NM_001032291.2:c.919C>T	NP_001027462.1:p.(Arg307Ter)	–	0.000840689	0	8.60E−06	1.29E−05	36	Neutral (91)	0.08066	0.0/0.09	HET	WT	HET	HET	HET	HET	HET	WT
	*KIF21A*^a^	NM_001173463.2:c.4324C>G	NP_001166934.1:p.(Leu1442Val)	–	0.000250627	0	8.24E−06	6.50E−06	25.9	Deleterious (87)	0.99321	0.0/1.12	HET	WT	HET	WT	HET	HET	HET	WT

In bold are individuals whom samples underwent WES trio analysis.

PredticSNP2 predictions were reported with the expected accuracy (%).

Genomic, transcript, and protein reference sequence identifiers are from NCBI database (GRCh37/hg19).

All NGS files were deposited in the SHGP repository [https://genomics.saudigenomeprogram.org/en/].

Variants LOVD IDs and other information are listed in Table S11.

*P* proband, *UM* unaffected mother, *AS* affected sibling, *UF* unaffected father, *US* Unaffected sibling, *1000G* 1000 Genomes, *SHGPdb* Saudi human genome program database.

^a^Mouse model(s) with behavioral and/or neurological phenotype has been documented for this gene in Mouse Genome Informatics database [http://www.informatics.jax.org], for details see Table S7.

^b^The same variant was detected in a homozygous state in one ASD case (unpublished data).

^c^Suspected ADHD case.

Almost half of the genes identified herein (15/31) have a mouse model with documented behavioral or neurological phenotype (Table S7). Among these genes *SLC9A9* was the only one with established mouse models of a neurodevelopmental disorder (ASD). This gene encodes a membrane protein localized in the late recycling endosomes, which are involved in the trafficking of neurotransmitters receptors and transporters. It is worth mentioning that we have screened all the genes listed in Table S1 for variants fulfilling our selection criteria to ensure that our pipeline had not missed any known causal or candidate variants in these genes. No candidate variants were flagged except the one that we have previously detected in *SLC9A9*. This gene was the only one overlapping with (IMAGE) [21].

### Biological pathways overrepresented in this study

In order to investigate which biological processes might be affected by the variants identified herein, we utilized two web-based bioinformatics tools (WebGestalt and Reactome). A list of diverse functional categories were flagged as statistically overrepresented within our final set of genes with validated variants (Table S8). By querying KEGG and GO databases, WebGestalt was able to identify the top ten functional categories under “pathway”, “biological process”, “cellular component” and “molecular function” that are significantly enriched in our gene list. Various biological themes were highlighted including “Insulin secretion”, “Glutamatergic, cholinergic, serotonergic, dopaminergic synapses”, “Circadian rhythm”, “Cell division”, “Pyruvate dehydrogenase complex”, and “Cytoskeleton organization”. In addition, Reactome identified the 25 most significant pathways, examples of which include; “Regulation of insulin secretion”, “Presynaptic function of Kainate receptors”, and “ Ca^2+^ pathway”. Interestingly, among all the enriched functional categories “Insulin secretion” was identified as a recurrent theme.

## Discussion

The role of rare SNVs has been much less investigated in ADHD, than in ASD, with no more than a handful of studies in which NGS was applied. These studies could be grouped into hypothesis-driven [14, 15], hypothesis- free [17–19] or a mix of both [16]. In the hypothesis-driven approach, NGS analysis was restricted to either a predefined set of ADHD candidate risk genes (IMAGE) [14], or gene sets curated from previous association studies (candidate genes/GWAS) after the application of specific criteria [15], or to a limited number of genes linked to ASD and intellectual disability (26 genes) [16]. With respect to sample size, all hypothesis-free studies were performed in a small number of cases/discovery-cohort (comprising 2–30 affected individuals), whereas larger number of samples (117–152 affected individuals) were included in the hypothesis-driven studies. Moreover, variant filtering criteria that was applied in the hypothesis-free investigations was relatively relaxed, not including either variant validation by an orthogonal method (Sanger sequencing or TaqMan assay) [18, 19], or variant functional impact prediction analysis [16, 17, 19].

In this study, we employed discovery-based approach coupled with our stringent variant filtering criteria to identify de novo and inherited rare (disease-relevant) candidate variants (Fig. 1). We have detected and validated 32 unique rare SNVs in 13/15 (86.6%) trios. For the majority of cases, at least two variants were found per proband. The presence of multiple candidate variants per case, is something that we and others have encountered in similar complex disorders (ASD and Parkinson’s disease) [22, 34, 35]. This reinforces the plausibility of a polygenic model whereby several genes/loci may harbor risk variants collectively contributing to the disease burden.

Assuming homozygous inheritance, we have previously analyzed all of the multi-incident families (Table S9) with apparently unaffected parents for regions of homozygosity shared only between affected individuals within or across families [36]. In our previous work the intention was to detect genomic regions of homozygosity that are likely to contain causal variants. However, in the current study our primary aim is to detect rare variants with predicted damaging effect regardless of the segregation pattern. It is perhaps unsurprising that none of the candidate genes revealed here in the multi-incident families existed in regions overlapping with what we have previously reported [36]. This is anticipated, given that genetic and allelic heterogeneity, reduced penetrance and variable expressivity are all factors known to influence the impact of genetic changes, an issue that has been increasingly recognized in complex disorders [33, 37]. Thus far, only four of the genes reported in ADHD NGS-based studies were replicated across cohorts from different descent (Table S10). Each was found to harbor distinct variants. However, whether or not they contribute to the disorder is yet to be discovered.

Our findings from both studies (here and [36]) are in keeping with the notion that ADHD does not conform to a monogenic model. Moreover, while homozygosity analysis may be less successful compared with WES in detecting rare variants with large effect size, it can be useful in highlighting genomic regions containing other types of common genetic events (structural, noncoding, or regulatory) that may act “ in aggregate” as modifiers [33].

*LRRK1* has been pointed out by our analysis as an interesting candidate gene. The LoF variant (c.2687-2A>G) detected within this gene was present in three out of the four affected individuals in F1. Recessive transmission of the alternative allele was observed in the three affected members (homozygous) while the rest of the family members (4th affected sibling along with the parents and the healthy siblings) were all heterozygous carriers. Under the assumption of a compound heterozygous transmission, we revisited the data generated from the CytoScan^TM^ HD platform for any CNVs affecting *LRRK1* in the 4th affected individual (with a heterozygous genotype). However, no copy number changes were detected in this gene. In spite of the reduced penetrance of this variant, it is the only one in this study that was present in more than two affected individuals (same allele and genotype) which was not shared with the unaffected family members. This variant affects a splice-acceptor site located between intron 19 and exon 20 (exons are numbered from 1 to 34 based on NM_024652/ ENST00000388948.3, GRCh37 genome build) which maps onto the ROC-COR bidomain of the protein [38]. However, assessment of its consequence on gene splicing pattern was not possible here due to the unavailability of RNA samples.

Thus far, osteosclerotic metaphyseal dysplasia, which is a rare form of skeletal dysplasia, is the only human condition thought to be caused by variants in *LRRK1* [39, 40]. Defective or depleted *LRRK1* has been shown to disrupt osteoclast normal function [39, 41]. Unfortunately, we could not determine whether the individuals homozygous for *LRRK1* variant had any form of bone abnormality due to the unavailability of relevant clinical and radiographic data.

Besides its role in bone homeostasis [41], *LRRK1* have been shown to play a role in Grb2-mediated EGFR endocytic trafficking [42]. EGFR signaling influences many cellular processes including proliferation and migration of neural progenitor cells [43, 44] and more recently has been identified as an essential regulator of axon branching in the developing *Drosophila* brain [45]. Proper axonal branching is crucial for the formation of functional neural circuits; disruption of which may underlie brain circuitry aberrations that have been documented in individuals with ASD and ADHD [46, 47]. However, whether EGFR signaling regulates axonal branch formation in the mammalian brain or not, is yet to be determined.

Of the biological themes revealed by our enrichment analysis, at least five categories overlapped with prior findings “Glutamatergic synapse” (40 genes reported in ADHDgene database), “Dopaminergic synapse” (20 genes reported in ADHDgene database), “Serotonergic synapse”, “Ca^2+^ signaling” (40 genes reported in ADHDgene database), and “Cytoskeleton organization”. These biological processes are central to brain function therefore it is unsurprising to find that they have been repeatedly described in the literature [19, 48, 49]. Moreover, functional categories pertaining to the cytoskeleton comprised the largest number of genes (7/31) followed by “Regulation of insulin secretion” (14 genes reported in ADHDgene database) (3/31). Interestingly, insulin signaling may indirectly be involved in normal brain function through its known role in regulating the activity of both excitatory and inhibitory synapses in addition to influencing the brain’s structural plasticity [50].

Small sample size is a limitation common to most of the published hypothesis-free ADHD genetic studies (including the present one) that can undermine the reliability of the produced findings. In fields such as human genetics of complex disorders, large-scale collaborative consortia are considered the gold standard approach for improving power and obtaining reliable and more generalizable results. However, extensive genetic and clinical heterogeneity not only across different cohorts/populations but also within families should be taken into consideration when this approach is employed. Another limitation of our study is that only the coding regions were interrogated for rare candidate variants, while variants existing outside our WES coverage area or those located in noncoding or regulatory regions were not possible to detect. In addition, the allele frequency of the CNVs identified here could not be determined due to the absence of data from matching normal controls.

In conclusion, our workflow revealed 32 unique rare variants in 31 different candidate genes most of which have not been previously described in ADHD. Even though our variant prioritization method is restricted to those with predicted deleterious effect, further functional and cellular analysis is essential to confirm their true biological consequences. The concept of a single causative variant being sufficient on its own to drive complex disorders like ADHD is no longer viable. Instead, an accumulating body of evidence indicates that multiple rare and common variants collectively contribute to the susceptibility to such disorders.

## Supplementary information

Supplementary text

Gene list curated for CNV analysis

Summary of the demographic data and relatedness analysis results

WES run quality metrics

Total variants detected in this study before applying filtering steps

List of genes with confirmed variants

Developmental and regional differential brain expression of the identified genes

Mouse model information for the identified genes.

Enrichment analysis results

Multi-incident families genetic findings from WES and homozygosity mapping

Summary of published NGS-based ADHD studies

LOVD information for the variants identified in this study
