# Supplementary material for: Whole exome sequencing in ADHD trios from single and multi-incident families implicates new candidate genes and highlights polygenic transmission
Source: Eur J Hum Genet. 2020 Apr 1;28(8):1098–110. doi: 10.1038/s41431-020-0619-7 (PMC7382449; doi:10.1038/s41431-020-0619-7)
Supplement: Supplementary file 1 — Supplementary text [file 41431_2020_619_MOESM1_ESM.docx]

**Supplementary Material**

**Whole exome sequencing in ADHD trios from single and multi-incident families implicates new candidate genes and highlights polygenic transmission**

Bashayer R. Al-Mubarak^1,2*^, Aisha Omar^1^, Batoul Baz^1,2^, Basma Al-Abdulaziz^1,3^, Amna I. Magrashi^1^, Eman Al Yemni^1,2^, Amjad Jabaan^2^, Dorota Monies^2,4^, Mohamed Abouelhoda^2,4^, Dejene Abebe^5^, Mohammad M. Ghaziuddin^6^, Nada A. Al Tassan^1,2*^

^*^Corresponding author

**Methods**

*Relatedness assessment*

We used two algorithms to compute the relationships between samples in each family. First, we utilized VCFtools package with option --relatedness (http://vcftools.sourceforge.net) ([1](#_ENREF_1)) to calculate the “unadjusted” Ajk relatedness statistic based on Yang et al ([2](#_ENREF_2)). Second, we computed normalized shared homozygosity between each pair of samples. Shared homozygosity is defined as the number of common homozygous variants between pairs of samples. We define normalized shared homozygosity as follows: Let S(M1,M2) define the shared homozygosity between family member M1 and M2. Then %Normalized Shared Homozygosity= 100 x shared homozygosity / ( S(M1,M2) + S(M1,M3) + S(M2,M3)).

For each method, we used a training data set and computed histograms of scores for related and non-related samples. Based on that, we used the following cutoffs: For the relatedness method, the relatedness is confirmed if the Ajk statistics is larger than 0.1. For the normalized shared homozygosity method, the relatedness is confirmed if the percent shared homozygosity is larger than 25%. Both methods showed consistent results.

*Functional enrichment analysis*

Two enrichment analysis tools were employed here to discover biological processes over-represented in our gene list. The first was WEB-based GEne SeT AnaLysis Toolkit (WebGestalt) [http://www.webgestalt.org] which serves as an integrated data mining system for functional enrichment analysis of large sets of genes ([3](#_ENREF_3), [4](#_ENREF_4)). This tool offers wide coverage of functional categories across diverse biological themes. This is achieved by extending the backend annotation data source to include not only publically and centrally-curated databases but also to include functional categories defined through computational network analysis. WebGestalt offers 3 main enrichment analysis methods, namely Over-Representation Analysis (ORA), Gene Set Enrichment Analysis (GSEA) and Network Topology-based Analysis (NTA). Here, enrichment analysis was carried out using ORA method developed for studies from which gene lists are directly generated ([5](#_ENREF_5)). Our gene list generated by the discovery-based approach was explored using two types of functional databases; 1) pathway (KEGG database), and 2) geneontology (biological process, molecular function and cellular component), in both settings human genome was used a reference. Moreover, the program employs two statistical tests (hypergeometric and the Fisher’s exact test) to identify enriched categories within the queried gene list and only the top 10 significantly (p-value<0.01) over-represented categories have been reported.

In addition to WebGestalt, we utilized the Reactome Knowledgebase [www.reactome.org] which functions as an archive for biomolecular pathways besides offering over-representation data analysis ([6](#_ENREF_6)). Reactome implements hypergeometric distribution statistical test in combination with Benjamani-Hochberg method to correct for false discovery rate. Of note, only most significant pathways were presented here.

**Supplementary Legends**

**Supplementary text**.

Contains extended methods (dox)

Table S1. Gene list curated for CNV analysis. (xls)

Table S2. Summary of the demographic data and relatedness analysis results. (xls)

Table S3. WES run quality metrics. (xls)

Table S4. Total variants detected in this study before applying filtering steps. (xls)

Table S5. List of genes with confirmed variants. (xls)

Table S6. Developmental and regional differential brain expression of the identified genes. (xls)

Table S7. Mouse model information for the identified genes. (xls)

Table S8. Enrichment analysis results. (xls)

Table S9. Multi-incident families genetic findings from WES and homozygosity mapping. (xls)

Table S10. Summary of published NGS-based ADHD studies. (xls)

Table S11. LOVD information for the variants identified in this study. (xls)

**References**

1. Danecek P, Auton A, Abecasis G, Albers CA, Banks E, DePristo MA, et al. The variant call format and VCFtools. *Bioinformatics*. 2011;27:2156-8.

2. Yang J, Benyamin B, McEvoy BP, Gordon S, Henders AK, Nyholt DR, et al. Common SNPs explain a large proportion of the heritability for human height. *Nat Genet*. 2010;42:565-9.

3. Wang J, Vasaikar S, Shi Z, Greer M, Zhang B. WebGestalt 2017: a more comprehensive, powerful, flexible and interactive gene set enrichment analysis toolkit. *Nucleic Acids Res*. 2017;45:W130-W7.

4. Zhang B, Kirov S, Snoddy J. WebGestalt: an integrated system for exploring gene sets in various biological contexts. *Nucleic Acids Res*. 2005;33:W741-8.

5. Khatri P, Sirota M, Butte AJ. Ten years of pathway analysis: current approaches and outstanding challenges. *PLoS Comput Biol*. 2012;8:e1002375.

6. Fabregat A, Jupe S, Matthews L, Sidiropoulos K, Gillespie M, Garapati P, et al. The Reactome Pathway Knowledgebase. *Nucleic Acids Res*. 2018;46:D649-D55.
